# Amino acid metabolomics and machine learning for assessment of post-hepatectomy liver regeneration

**DOI:** 10.3389/fphar.2024.1345099

**Published:** 2024-05-24

**Authors:** Yuqing Yan, Qianping Chen, Xiaoming Dai, Zhiqiang Xiang, Zhangtao Long, Yachen Wu, Hui Jiang, Jianjun Zou, Mu Wang, Zhu Zhu

**Affiliations:** ^1^ School of Basic Medicine and Clinical Pharmacy, China Pharmaceutical University, Nanjing, China; ^2^ Department of Clinical Pharmacology, Nanjing First Hospital, Nanjing Medical University, Nanjing, China; ^3^ The First Affiliated Hospital, Department of Hepatobiliary Surgery, Hengyang Medical School, University of South China, Hengyang, Hunan, China; ^4^ Hengyang Medical School, University of South China, Hengyang, Hunan, China; ^5^ Department of Pharmacy, Nanjing First Hospital, China Pharmaceutical University, Nanjing, China; ^6^ The NanHua Affiliated Hospital, Clinical Research Institute, Hengyang Medical School, University of South China, Hengyang, Hunan, China

**Keywords:** hepatectomy, metabolomics, machine learning, liver regeneration, non-alcoholic steatohepatitis (NASH)

## Abstract

**Objective:**

Amino acid (AA) metabolism plays a vital role in liver regeneration. However, its measuring utility for post-hepatectomy liver regeneration under different conditions remains unclear. We aimed to combine machine learning (ML) models with AA metabolomics to assess liver regeneration in health and non-alcoholic steatohepatitis (NASH).

**Methods:**

The liver index (liver weight/body weight) was calculated following 70% hepatectomy in healthy and NASH mice. The serum levels of 39 amino acids were measured using ultra-high performance liquid chromatography–tandem mass spectrometry analysis. We used orthogonal partial least squares discriminant analysis to determine differential AAs and disturbed metabolic pathways during liver regeneration. The SHapley Additive exPlanations algorithm was performed to identify potential AA signatures, and five ML models including least absolute shrinkage and selection operator, random forest, K-nearest neighbor (KNN), support vector regression, and extreme gradient boosting were utilized to assess the liver index.

**Results:**

Eleven and twenty-two differential AAs were identified in the healthy and NASH groups, respectively. Among these metabolites, arginine and proline metabolism were commonly disturbed metabolic pathways related to liver regeneration in both groups. Five AA signatures were identified, including hydroxylysine, L-serine, 3-methylhistidine, L-tyrosine, and homocitrulline in healthy group, and L-arginine, 2-aminobutyric acid, sarcosine, beta-alanine, and L-cysteine in NASH group. The KNN model demonstrated the best evaluation performance with mean absolute error, root mean square error, and coefficient of determination values of 0.0037, 0.0047, 0.79 and 0.0028, 0.0034, 0.71 for the healthy and NASH groups, respectively.

**Conclusion:**

The KNN model based on five AA signatures performed best, which suggests that it may be a valuable tool for assessing post-hepatectomy liver regeneration in health and NASH.

## 1 Introduction

Liver regeneration is a biological process that occurs under moderate stimulation, such as surgery and injury. Parenchymal and mesenchymal cells, as well as several molecules including metabolites and cytokines, participate in this process (Lorente et al., 2020). The process of liver regeneration consists of three stages: the priming phase (0–6 h), proliferation phase (12–72 h), and termination phase (96–168 h) (Sergeeva et al., 2020). Due to high regeneration capacity, a healthy liver can achieve functional compensation within several weeks after 80% of the liver is resected (Yang et al., 2020). Owing to the above characteristics, partial hepatectomy has been successfully used as the surgical modality for benign and malignant liver diseases, including hepatic hemangioma, hepatolithiasis, and liver cancer (Ahmed et al., 2016; Michalopoulos, 2017; Abu Rmilah et al., 2019). However, the capacity of liver regeneration in patients with chronic liver disease is typically worse than that of the healthy population (de Meijer et al., 2010). The assessment of liver regeneration following hepatectomy is affected by the severity of chronic liver disease, which poses a considerable challenge for disease treatment and prognosis (Ascha et al., 2010; Guglielmi et al., 2012; Geh et al., 2021).

Non-alcoholic steatohepatitis (NASH) is one of the most common chronic liver diseases globally, and it is estimated that there will be 350 million patients worldwide by 2030 (Konerman et al., 2018). NASH, which is characterized pathologically by steatosis, inflammation, and hepatocyte injury with/without fibrosis, may ultimately progress to cirrhosis or even hepatocellular carcinoma (Liu et al., 2016). As liver regeneration is significantly affected, the post-hepatectomy liver function, overall incidence of complications, and mortality rate are increased considerably in NASH patients (de Meijer et al., 2010; Reddy et al., 2012). Therefore, the evaluation of post-hepatectomy liver regeneration in NASH is a clinical issue that urgently requires a solution.

Currently, computed tomography and magnetic resonance imaging are widely used in clinical practice for measuring post-hepatectomy residual liver volume to evaluate liver regeneration (Zamboni et al., 2008; Luciani et al., 2012; Soyer et al., 2012). However, the imaging investigations not only result in erroneous or overestimated actual liver volume, but also bring about difficulties in transporting and caring for patients undergoing hepatectomy (D'Onofrio et al., 2014). Therefore, it is crucial to develop an accurate and convenient assessment method for post-hepatectomy liver regeneration.

Metabolomics, a general method in systems biology that measures metabolites to discover specific metabolomes, plays a crucial role in biomarker screening for medical diagnosis and assessment (Di Minno et al., 2022). Machine learning (ML) is a core technique that uses artificial intelligence in which big data calculation is utilized to generate empirical models. ML has substantially improved the prediction of disease etiology, outcome, and prognosis, and it has gradually become an essential component of modern medical decision-making (Deo, 2015; Handelman et al., 2018). Studies combining ML and metabolomics have resulted in more precise diagnose of cancer, cerebro-cardiovascular disease, diabetes, and COVID-19 (Bifarin et al., 2021; Jung et al., 2021; Bratulic et al., 2022; Galal et al., 2022; Ji et al., 2022). Sun et al. (2021) employed non-targeted metabolomics in combination with ML and identified four amino acids (AAs) including ornithine, phenylalanine, lysine, and 2-hydroxybutyric that can assess the liver index of healthy mice during liver regeneration. AAs are the precursors of many important biomolecules, and their metabolism is closely associated with liver regeneration (Wu, 2009). However, it is still unclear whether the combination of amino acid (AA) metabolomics with ML can assess liver regeneration in different liver conditions, such as NASH.

In this study, we combined AA metabolomics with ML to identify the relationship between post-hepatectomy serum AA levels and liver index for different liver conditions. Our study would provide new insights for assessing liver regeneration following hepatectomy.

## 2 Materials and methods

### 2.1 Experimental animals and materials

Seventy-two 6–8-week-old male C57BL/6J mice weighing 18–20 g were used in this study (Hunan SJA Laboratory Animal Co., Ltd., Changsha, China). They were housed in rooms at a constant temperature of 22°C ± 2°C, relative humidity of 50% ± 10%, and light cycle of 12 h/d (8:00 to 20:00). The mice were given *ad libitum* access to water and food.

Acquity ultra-performance liquid chromatography system, Xevo TQ-S Micro mass spectrometer, Masslynx 4.1 and Acquity UPLC HSS T3 Column (2.1 × 100 mm, 1.8 μm) (Waters, Milford, MA, United States), methanol (Sinopharm, Shanghai, China), formic acid (CNW Technologies GmbH, Dusseldorf, Germany), acetonitrile (Fisher Scientific, Waltham, MA, United States), and ultrapure water (Merck, Darmstadt, Germany) were utilized as purchased. The internal standard (IS) for the stable isotope label was purchased from Cambridge Isotope Laboratories (Andover, MA, United States). All commercially available chemicals used in this study were analytical-grade reagents.

### 2.2 Experimental methods

#### 2.2.1 Construction of 70% hepatectomy mice model under different liver conditions

Seventy-two mice were randomized into healthy (*n* = 36) and NASH group (*n* = 36). The healthy group was given a standard diet for 12 weeks. The NASH group was given the Gubra-Amylin-NASH diet (D09100310, 40% fat, 22% fructose, and 2% cholesterol) for 12 weeks (Boland et al., 2019; Fujisawa et al., 2021). Then, the left and middle lobes of the liver were resected according to our previous studies (Lei et al., 2022; Dai et al., 2023; Wang et al., 2023). The mice were anesthetized and sacrificed at 0 h (sham group), 6, 24, 48, 72, and 168 h following the 70% hepatectomy (six mice at each time point). The right lobe and blood were harvested, and the liver index (liver weight/body weight) was calculated. The tissue specimens were embedded in paraffin, sectioned, and stained with hematoxylin and eosin (H&E). Immunohistochemistry using an anti-PCNA antibody (Santa Cruz Biotechnology, sc-56, 1:200 dilution) was performed to assess hepatocyte proliferation. The blood was allowed to stand for 30 min at room temperature before centrifugation at 3,000 rpm and maintained at 4°C for 5 min. The serum was collected and stored at −80°C. The animal experiments, which conformed to internationally recognized institutional animal care and use guidelines, were approved by the Institutional Animal Care and Use Committee of the University of South China.

#### 2.2.2 Sample extraction

We collected 50 μL serum samples, and 500 μL of methanol: water (50: 50 v/v) was added. The solution was vortexed for 5 min before centrifugation at 12,000 rpm and maintained at 4°C for 10 min. Then, 250 μL of supernatant was collected, and 20 μL of the IS solution was added. The solution was centrifuged at 12,000 rpm and maintained at 4°C for 5 min. Afterward, 2 μL of the supernatant was collected for metabolomics analysis of AA. Quality control samples (QC) are a mixture of the test samples, with 5 μL taken from each sample for the mixture, followed by repeated testing.

#### 2.2.3 UPLC–MS/MS analysis

The chromatography conditions were as follows. Mobile phase A: 0.1% formic acid aqueous solution; mobile phase B: acetonitrile. The flow rate was 0.5 mL/min. The gradient conditions for the mobile phase were as follows. Mobile phase A:B: 0 min 98:2 (v/v), 4 min 78:22 (v/v), 4.1 min 5:95 (v/v), 4.4 min 0:100 (v/v), 4.6 min 98:2 (v/v), and 6 min 98:2 (v/v). The sample was placed in a 4°C autosampler. The column temperature was 45°C. The flow rate was 250 μL/min, and the sample volume was 2 μL. Mass spectrometry was performed as follows. A triple quadrupole linear ion trap mass spectrometer was used. The spectrometer was equipped with an ESI ion source and used in positive ion mode. The ESI source parameters were as follows. The source temperature was 350°C, and the voltage was 3000 V. We optimized the declustering potential and collision energy scanning detection of the ion pairs. Supplementary Table S1 shows the detailed conditions for these measurements.

#### 2.2.4 Metabolomics analysis

Masslynx (V4.1, Waters, United States) software was used for peak extraction of the raw multiple reaction monitoring data to obtain the ratios of various AA peak areas to the IS peak area. The content was calculated based on the standard curve. All quantitative metabolomics data were log10-transformed and unit variance scaling. SIMCA-P software (V14.1, Umetrics, Sweden) was used for orthogonal partial least squares discriminant analysis (OPLS-DA) to screen for metabolites with variables of importance in the project (VIP) > 1. Metabolites with VIP >1 and *p* < 0.05 were considered differential metabolites. In SIMCA-P, global metabolic differences were observed in the healthy, NASH, and QC groups using principal component analysis (PCA), whereas metabolic differences in the healthy and NASH groups at different time points were analyzed using OPLS-DA. Afterward, the MetaboAnalyst (www.metaboanalyst.ca) website was used for pathway enrichment analysis of the differential metabolites.

#### 2.2.5 Five ML models to assess the liver index

Five machine learning models, including least absolute shrinkage and selection operator model (LASSO), random forest model (RF), K-nearest neighbor model (KNN), support vector regression model (SVR), and extreme gradient boosting model (XGB), were used to assess the liver index. The hyperparameters of each model were adjusted through the grid search algorithm and 5-fold cross-validation. The contribution of AAs to the model was interpreted using SHapley Additive exPlanations (SHAP) algorithm (Lundberg and Lee, 2017). The mean absolute error (MAE), root mean square error (RMSE), and coefficient of determination (*R* ^2^) were used to evaluate model performance. The ML models were built using the “sklearn 1.0.2” package, and SHAP algorithm was performed using the “shap 0.41.4” package in Python (version 3.9.13).

#### 2.2.6 Statistical analysis

AAs with more than 20% missing data were excluded, and missing <20% were imputed using KNN Impute (Troyanskaya et al., 2001). One-way ANOVA or Kruskal-Wallis H test was used, followed by pairwise comparisons between different time points, then adjusted by the Benjamini-Hochberg method to control the False Discovery Rate. The Wilcoxon test was used for statistical analysis of the MAE, RMSE, and *R* ^2^ of the ML models. The correlation between AA concentration and liver index was presented by Pearson’s correlation coefficient. Liver index of healthy and NASH groups at the same time points were tested using independent samples t-test. R Project (version 4.1.2) was used for data analysis. *p* < 0.05 was considered statistically significant.

## 3 Results

### 3.1 Liver pathological characteristics and regeneration

**FIGURE 1:**
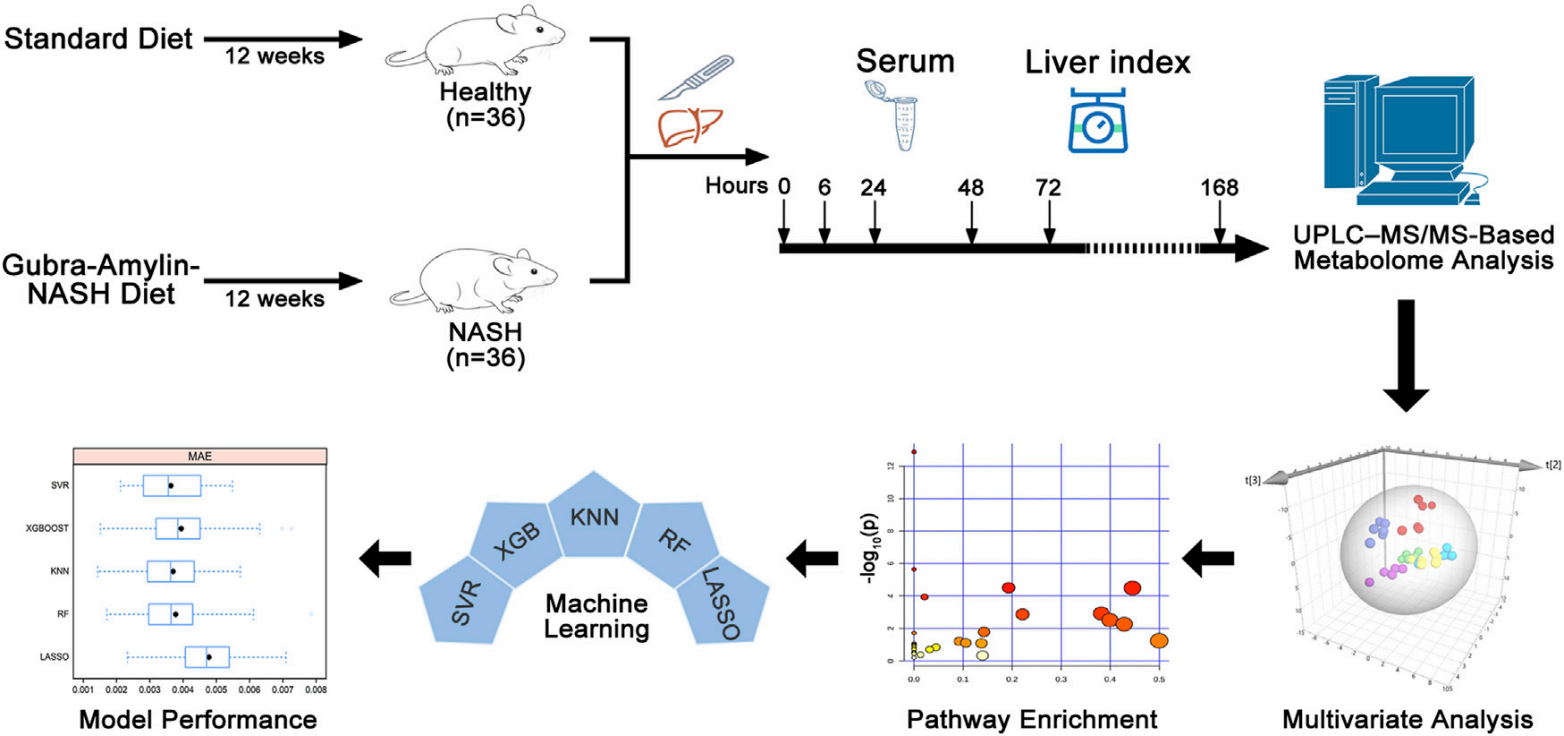
Study design and data analysis workflow.

**FIGURE 2:**
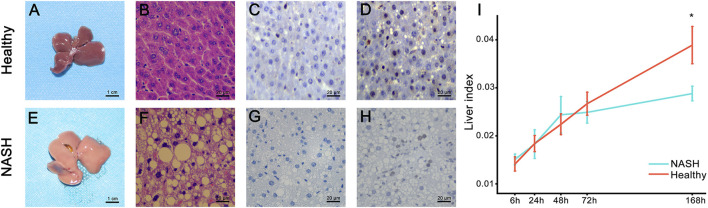
Pathology presentation of the liver. **(A,E)** Gross presentation. **(B,F)** HE staining. Postoperative PCNA expression at **(C,G)** 6 h and **(D,H)** 72 h. **(I)** Comparison of the liver index after PH between the healthy (red line) and NASH groups (blue line). *The difference in the liver index between the two groups was statistically significant (*p* < 0.05). Abbreviations: NASH, non-alcoholic steatohepatitis; HE, hematoxylin-eosin; PH, partial hepatectomy.

The study design is illustrated in Figure 1. We successfully constructed 70% partial hepatectomy (PH) models in healthy and NASH mice. Two pathologists confirmed the pathological characteristics of NASH in a blinded manner by macroscopic and microscopic findings, including light pink, soft surface and blunt edge as well as cytoplasmic round lipid droplets, balloon-like degeneration, and inflammatory cell infiltration (Figures 2A, B, E, F. PCNA immunohistochemistry of liver tissues showed that the PCNA positivity rates at 6 h and 72 h after hepatectomy in the NASH group was significantly lower than that in the healthy group (Figures 2C, D, G, H). We used the liver index to evaluate liver regeneration in the PH model for both groups. At 6, 24, 48 and 72 h time points, there seems to be no significant difference in the liver index between the two groups. However, the liver index in the NASH group is significantly lower than that in the healthy group at 168 h (*p* < 0.05) (Figure 2I, Supplementary Table S2).

### 3.2 Metabolomics data analysis

**FIGURE 3:**
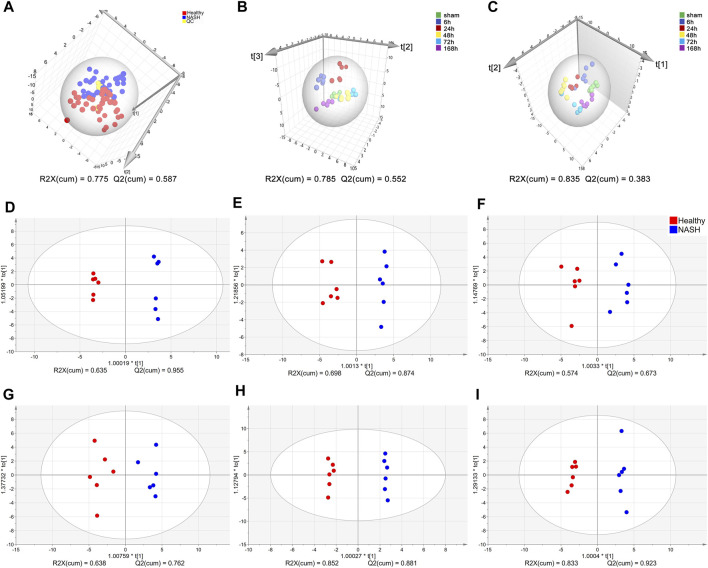
Multivariate statistical analysis of AA concentrations. **(A)** PCA score plots of healthy, NASH and QC groups. **(B)** OPLS-DA score plot at each time point in the health group. **(C)** OPLS-DA score plot at each time point in the NASH group. OPLS-DA score plot differentiating healthy from NASH in **(D)** 0 h, **(E)** 6 h, **(F)** 24 h, **(G)** 48 h, **(H)** 72 h, **(I)** 168 h. Each point represents an AA metabolic profile of a biological sample. The axes are the principal components of the models, and R2X represents the rate of explanation of the model in the principal component direction for the original data. Abbreviations: AA, amino acid; PCA, principal component analysis; NASH, non-alcoholic steatohepatitis; QC, control samples; OPLS-DA, orthogonal partial least squares discriminant analysis.

A total of 39 AAs were quantified via tandem mass spectrometry (MS/MS) in this experiment (see Supplementary Data Sheet). Due to a significant number of missing values, bAib was excluded from the analysis. The concentration changes of the remaining 38 AAs in the healthy and NASH groups are presented in Supplementary Figure S1. The QC samples were clustered well in the PCA score plots (Figure 3A), indicating stable instrument detection and good reproducibility throughout the experiment. And there is a small overlap in the sample regions between the healthy and NASH groups, indicating significant metabolic differences between the two groups. We performed OPLS-DA to obtain an overview of AA metabolic data after PH in healthy and NASH groups (Figures 3B–C). The scatter plots of the healthy and NASH groups were similar. The data at 6, 24, 48, and 72 h time points were clearly separated from those of 0 h (sham), whereas the data at 168 h were close to those at 0 h. This showed that PH induced AA metabolism changes at the priming phase and proliferation phase of liver regeneration. Furthermore, the supervised OPLS-DA model revealed clear separation of samples between the healthy and NASH groups at each time point, indicating significant metabolic differences between the two groups (Figures 3D–I).

In healthy group, 11 differential AAs with VIP >1 and *p* < 0.05 were obtained from the screening, including Met, Asn, Hyl, Hcit, Tyr, Pro, 3MHis, Ala, Hyp, Ser, and Val. In NASH group, 22 differential AAs with VIP >1 and *p* < 0.05 were obtained from the screening, such as Asa, Sar, Hyl, bAla, Arg, Hyp, Val, His, Abu, Leu, 1MHis, Tyr, Ile, Asn, GABA, Asp, Cys, Tau, Gly, Gln, EtN, and Thr (see Supplementary Tables S3A, B). The above-mentioned differential AAs were used to build machine learning models.

### 3.3 Pathway enrichment analysis

**FIGURE 4:**
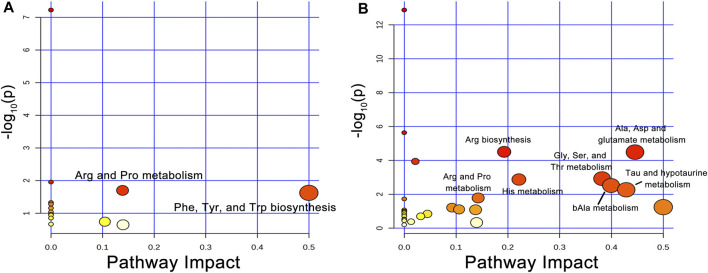
Disturbed AA metabolic pathways during liver regeneration in healthy and NASH groups. **(A)** Arg and Pro metabolism; Phe, Tyr, and Trp biosynthesis were disturbed metabolic pathways following PH in the healthy group, **(B)** Arg biosynthesis; Ala, Asp and glutamate metabolism; Gly, Ser, and Thr metabolism; His metabolism; bAla metabolism; Tau and hypotaurine metabolism; Arg and Pro metabolism were disturbed metabolic pathways after PH in the NASH group. Abbreviations: AA, amino acid; PH, partial hepatectomy; NASH, non-alcoholic steatohepatitis.

To identify the metabolic pathways that were significantly disturbed during liver regeneration, we used an online tool, MetaboAnalyst (http://www.metaboanalyst.ca), for analysis of the AAs that had a VIP >1. The VIP AAs of the two groups were mapped onto KEGG metabolic pathways for pathway enrichment analysis. In this study, the pathways with threshold >0.1 and *p* < 0.05 were considered the central metabolic pathways and presented as a bubble plot (Figures 4A, B). Arg and Pro metabolism and the biosynthesis of Phe, Tyr, and Trp were the most affected metabolic pathways in healthy group. Arg biosynthesis; Ala, Asp, and glutamate metabolism; Gly, Ser, and Thr metabolism; His metabolism; bAla metabolism; Tau and hypotaurine metabolism; and Arg and Pro metabolism were the most affected pathways in NASH group. Supplementary Tables S4A, B shows the overview of the enrichment analysis of the pathway-related metabolite sets.

### 3.4 Comparison and optimization of ML models

#### 3.4.1 Selection of AA signatures and comparison of machine learning models

**FIGURE 5:**
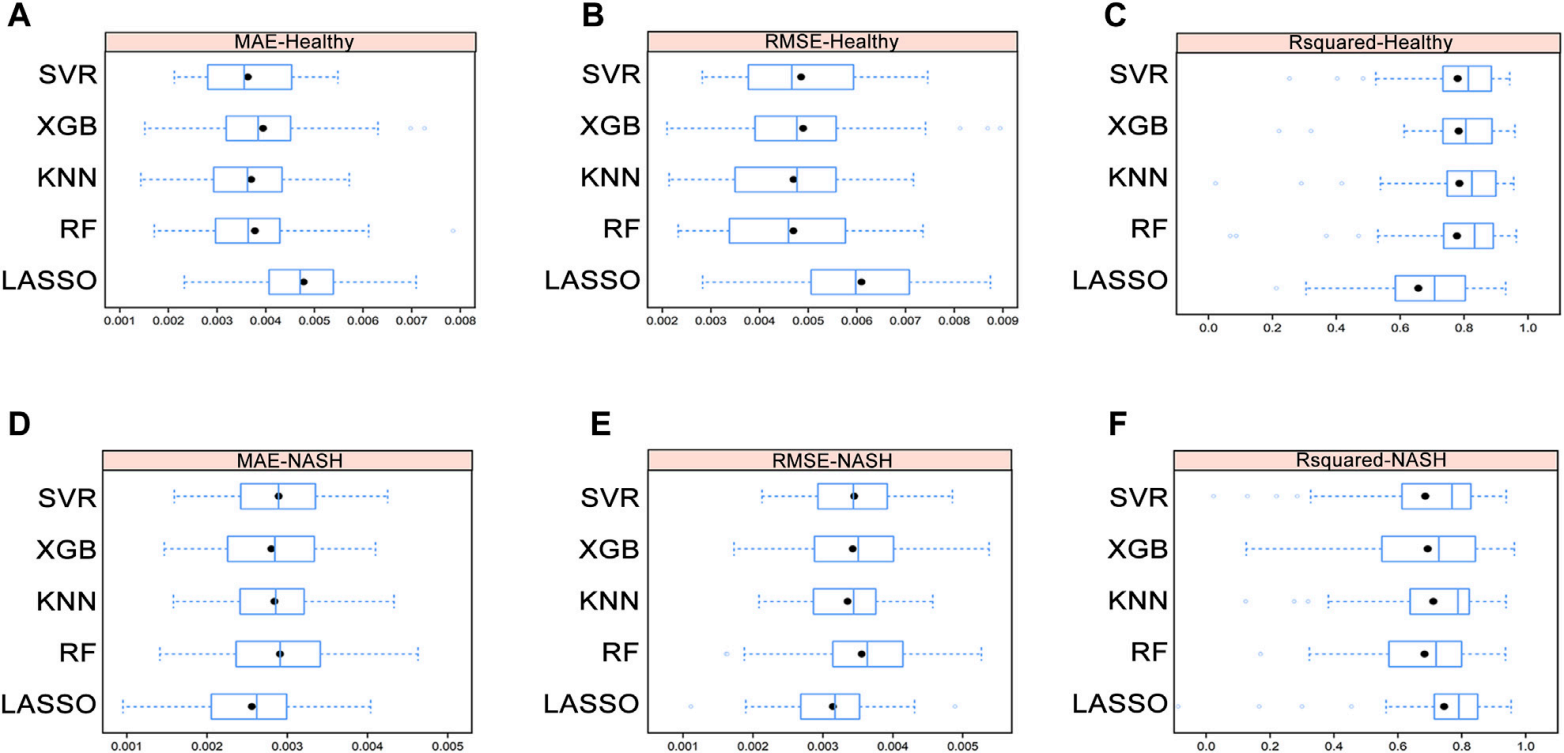
Average MAE **(A)**, RMSE **(B)**, and *R* ^2^ **(C)** on ten repeated 5-fold cross-validation of five machine learning models for assessment of the liver index in the healthy group. Average MAE **(D)**, RMSE **(E)**, and *R* ^2^ **(F)** on ten repeated 5-fold cross-validation of five machine learning models for assessment of the liver index in the NASH group. Abbreviations: MAE, mean absolute error; RMSE, root mean square error; *R* ^2^, coefficient of determination; NASH, non-alcoholic steatohepatitis.

We used five ML models, including LASSO, RF, KNN, XGB, and SVR, to construct assessment models for the liver index in healthy and NASH groups. The importance of AAs in each model was determined by the SHAP algorithm (see Supplementary Data Sheet). To minimize the number of metabolites for accurate measurement, five top-ranked AAs were used to build the optimization model. The hyperparameters of the optimization model are shown in Supplementary Tables S5A, B. We performed 5-fold cross-validation ten times on the datasets and calculated the MAE, RMSE, and *R* ^2^ to evaluate model performance (see Figures 5A–F and Supplementary Tables S6A, B).

In healthy group, the Wilcoxon test showed that there were no differences in MAE, RMSE, and *R* ^2^ among the RF, KNN, XGB, and SVR models, while the KNN model had the lowest mean RMSE and highest mean *R* ^2^. In NASH group, the Wilcoxon test demonstrated that the KNN model still performed well despite no significant differences in MAE, RMSE, and *R* ^2^ among the five models (see Supplementary Tables S7A–D). Furthermore, the correlation between the differential AAs and the liver index indicated that AA signatures included in the KNN model were strongly correlated with the liver index (Supplementary Figures S2A, B and Supplementary Tables S8A, B). Therefore, KNN model was selected as the final assessment model.

#### 3.4.2 Correlation of AA signatures with liver index and performance of KNN models

**FIGURE 6:**
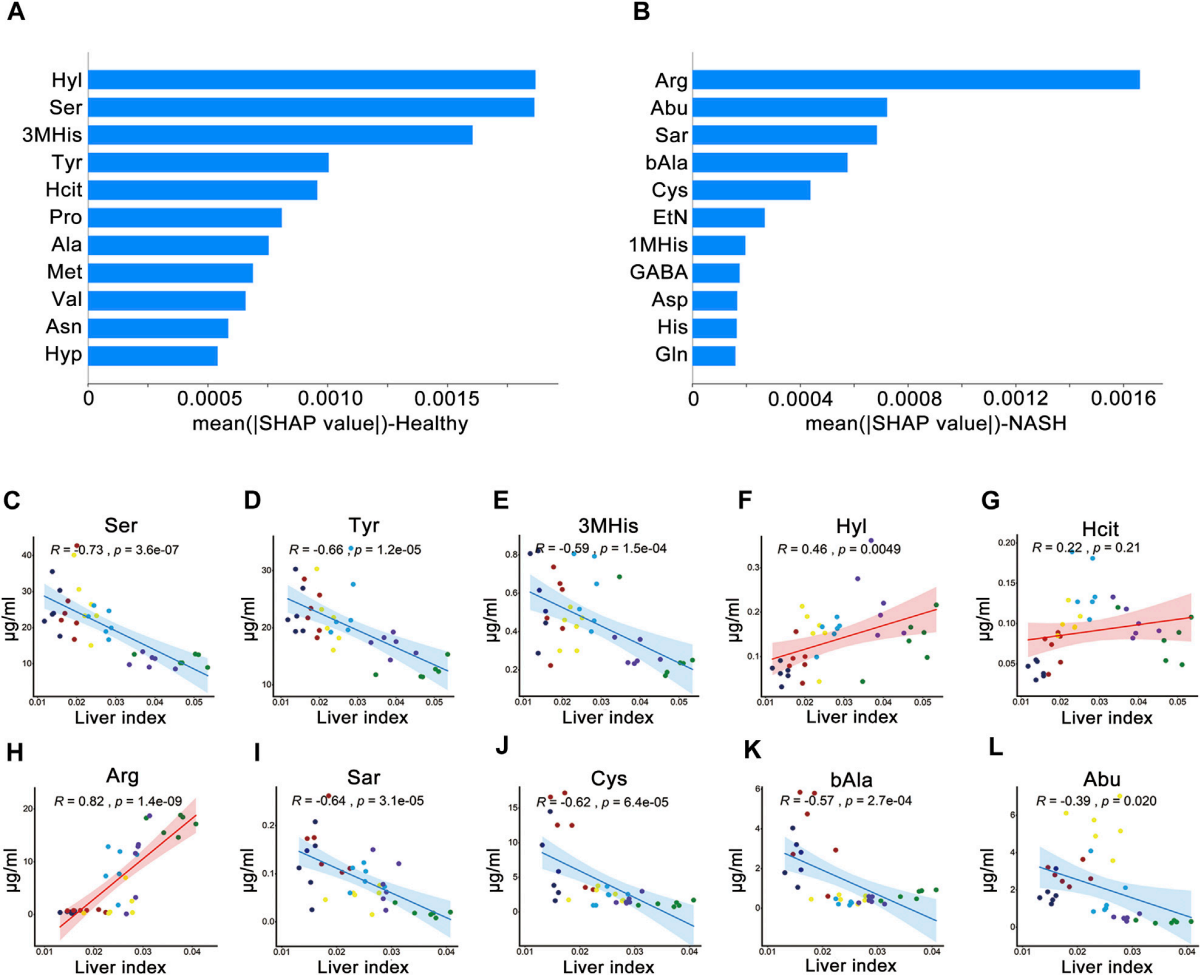
SHAP analysis and Pearson’s correlation coefficients between the liver index and AAs. Importance ranking of the top 11 AAs of the ML model for **(A)** health group and **(B)** NASH group. Correlation of liver index with **(C)** Ser, **(D)** Tyr, **(E)** 3MHis, **(F)** Hyl, and **(G)** Hcit in healthy group. Correlation of liver index with **(H)** Arg, **(I)** Sar, **(J)** Cys, **(K)** bAla, and **(L)** Abu in NASH group. Abbreviations: SHAP, SHapley Additive exPlanations algorithm; AAs, amino acids; ML, machine learning; NASH, non-alcoholic steatohepatitis; R, Pearson’s correlation coefficient.

**FIGURE 7:**
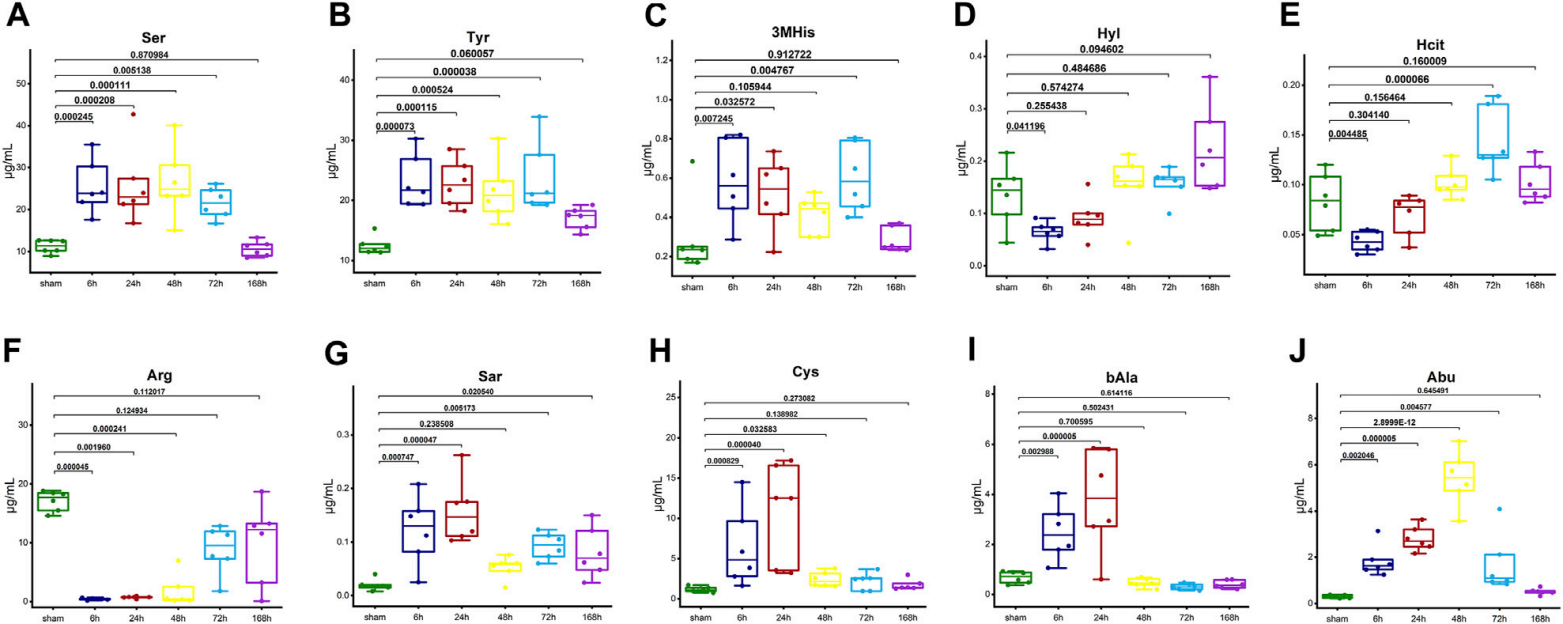
The box plots illustrate the trends in concentration changes of AA signatures. **(A)** Ser, **(B)** Tyr, **(C)** 3MHis, **(D)** Hyl, and **(E)** Hcit in the healthy group. **(F)** Arg, **(G)** Sar, **(H)** Cys, **(I)** bAla, and **(J)** Abu in the NASH group. The graphs show the *p*-value (One-way ANOVA or Kruskal-Wallis H test followed by pairwise comparisons) of each time point compared with sham. Abbreviations: AA, amino acid; NASH, non-alcoholic steatohepatitis.

Figures 6A, B shows the importance of AAs in the KNN model. The AA signatures included in the KNN model for healthy group were Hyl, Ser, 3MHis, Tyr, and Hcit. Notably, Ser, 3MHis, and Tyr were significantly negatively correlated with the liver index, whereas Hyl was significantly positively correlated with the liver index (Figures 6C–G). Moreover, the AA signatures included in the KNN model for NASH group were Arg, Abu, Sar, bAla, and Cys. Likewise, Abu, Sar, Cys, and bAla were significantly negatively correlated with the liver index, while Arg was significantly positively correlated with the liver index (Figures 6H–L). The changes in the concentration of these AA signatures are exhibited in Figure 7.

Cross-validation showed that in healthy group, the KNN model had a MAE of 0.0037 ± 0.0010, a RMSE of 0.0047 ± 0.0013, and a *R* ^2^ of 0.79 ± 0.17, and in NASH group, it had a MAE of 0.0028 ± 0.0006, a RMSE of 0.0034 ± 0.0009, and a *R* ^2^ of 0.71 ± 0.18.

## 4 Discussion

Based on a 70% hepatectomy model in mice under healthy and NASH conditions, we combined AA metabolomics and ML to explore the biological correlation between AA metabolism and liver regeneration. Using differential AAs as the assessment signature, we constructed five ML models, including LASSO, RF, KNN, SVR, and XGB. Remarkably, the KNN model performed best in the healthy group (MAE = 0.0037, RMSE = 0.0047, *R* ^2^ = 0.79, and the NASH group (MAE = 0.0028, RMSE = 0.0034, *R* ^2^ = 0.71). This study has successfully measured the liver index during liver regeneration in different liver conditions, which offers new insights for the assessment of liver regeneration after hepatectomy.

Some recent metabolomics studies have shown that AA metabolism is closely associated with post-hepatectomy liver regeneration. Zhao et al. (2020) constructed a rat PH model and employed gas chromatography–mass spectrometry to analyze postoperative serum samples and found that AA metabolism pathways showed the most significant changes in liver regeneration. Carril et al. (2020) used liquid chromatography–mass spectrometry to analyze post-hepatectomy liver tissue samples from NASH mice and found that AA metabolism changed significantly during the termination phase of liver regeneration. Additionally, among several metabolites, Sun et al. (2021) identified AAs as the most optimal evaluated markers for the liver index. To summarize, both previous research and our study demonstrated that AA metabolism is closely related to liver regeneration after hepatectomy, and AAs represent a valuable biomarker for assessing liver regeneration.

In this study, we found that Arg and Pro metabolism were commonly disturbed metabolic pathways during liver regeneration in healthy and NASH groups. Arg is the precursor of Pro and hydroxyproline and can be converted to polyamine through ornithine. Polyamine is associated with cell growth and can stimulate biosynthesis (Canellakis et al., 1985). Yang et al. (2020) constructed a rat PH model, carried out combined proteome and metabolome analysis, and found that Arg biosynthesis correlates with the priming phase of liver regeneration. Additionally, Bottiglieri et al. (2022) performed metabolome analysis using serum and urine samples from recipients after living donor liver transplantation, and they found that the ratio of dimethylarginine to Arg is related to hepatic function recovery during liver regeneration. Therefore, we hypothesized that the Arg and Pro metabolic pathways may be conserved metabolic pathways in post-hepatectomy liver regeneration under different liver conditions.

AA signatures assessing liver regeneration in the healthy group were identified. Ser, 3MHis, Tyr, Hyl, and Hcit content varied significantly during liver regeneration and greatly contributed to model performance. Serine is a precursor for synthesizing many AAs, nucleotides, phospholipids, and choline. In this study, Ser content changed markedly at 6 h following PH. Similarly, Xu and Chang (2008) found that Ser content changed significantly at 48 h after PH in rats. Thus, it is evident that Ser content changes primarily in the early stage of liver regeneration. In addition, 3MHis is an AA that participates in the assembly of skeletal muscle contraction proteins. Kajiura et al. (2019) conducted a post-hepatectomy metabolome analysis using serum and urine samples from hepatocellular carcinoma patients and found that 3MHis significantly increased 3 days after surgery. Moreover, there is an increase in Tyr biosynthesis and metabolism during liver regeneration (Gasman et al., 1997; Xu and Chang, 2008). Likewise, the metabolic level of Hyl and Hcit showed significant changes during liver regeneration and play an important role in liver index assessment. Based on a 70% hepatectomy model in healthy mice, Sun et al. (2021) combined metabolomics with ML and found that AA signatures including ornithine, phenylalanine, lysine, and 2-hydroxybutyric are capable of measuring liver regeneration, which differs from the results of our study. The discrepancy may be attributed to variations in study design, including time point selection, AA profile tested, and mass spectrometry detection method. However, it is noteworthy that Ser and Tyr content showed a significant negative correlation with liver index in our study, which is consistent with Sun’s study. In addition, although beyond the AA profile detected in their study, 3MHis, Hyl, and Hcit showed assessing capability. Therefore, our study will provide a new experimental basis for further exploration of assessment for liver regeneration.

To the best of our knowledge, this is the first assessment model for liver regeneration in the context of NASH. We identified five AAs signatures including Arg, Sar, bAla, Abu, and Cys. Firstly, Arg is a nitric oxide (NO) precursor and participates in the synthesis and metabolism of various nutrients. Previous studies showed that both Arg and NO increased significantly during liver regeneration (Minin et al., 2005; Xu and Chang, 2008). NO is an essential regulator of hepatocyte proliferation in liver regeneration, which not only promotes liver regeneration through vasodilatation and increased hepatic artery blood flow, but also enhances the anti-apoptotic ability of regenerating liver by preventing TNF-α-mediated activation of proapoptotic caspase-3 (Pahlavan et al., 2006; Xu and Chang, 2008; Mei and Thevananther, 2011). Secondly, Sar is an N-alkylglycine, intermediate and byproduct in the synthesis and degradation of Gly (Mei and Thevananther, 2011). Ito et al. (2008) discovered that Gly improves regeneration from severe ischemia/reperfusion injury in the liver after PH and is beneficial for the prognosis of hepatectomy patients. Thirdly, bAla is a neurotransmitter or hormone regulator that can regulate *in vivo* metabolism. Sun et al. (2021) found that bAla is associated with liver regeneration, which demonstrated a common metabolic pathway as in our study. Lastly, Abu, a byproduct of Cys biosynthesis from cystathionine, can modulate glutathione (GSH) homeostasis (Irino et al., 2016; Sun et al., 2021). GSH plays a pivotal role as a major antioxidant, and its depletion makes the liver vulnerable to oxidative stress and predisposes it to the progression of chronic liver disease such as NASH (Jung et al., 2012; Jung, 2015). Several studies reported significant changes in GSH and Cys after PH, further supporting our findings (Huang et al., 1998; Jung et al., 2013). Using multivariate analysis of OPLS-DA, correlation analysis and SHAP algorithm, we identified the above AA signatures which showed a significant correlation with liver index and contributed most to the measurement. Thus, our findings will provide new insights for assessing liver regeneration on the background of NASH.

However, there were some limitations in this study. First, biomarker reproducibility is challenging for disease diagnosis and assessment in studies. Second, it is insufficient to use AA metabolomics to characterize the liver regeneration process, while proteomics, genomics, and multiomics studies are also required. Finally, although our model showed good performance in mice, there are variations between animals and humans. In the future, large-scale, multiomics studies in a spectrum of chronic liver diseases should be conducted to enhance the evaluation accuracy of post-hepatectomy liver regeneration.

## 5 Conclusion

By studying the process of liver regeneration and AA metabolism in healthy and NASH mice following PH, we found that Arg and Pro metabolism were commonly disturbed metabolic pathways in both groups during liver regeneration. In healthy mice, Hyl, Ser, 3MHis, Tyr, and Hcit were identified as signature AAs related to liver regeneration. Arg, Abu, Sar, bAla, and Cys were determined as signature AAs associated with liver regeneration in NASH mice. The KNN model, which was developed to assess the liver index of two groups at different time points, showed satisfactory assessment performance. These findings would provide new insights for assessing liver regeneration after hepatectomy.

## Data Availability

The original contributions presented in the study are included in the article/Supplementary Material, further inquiries can be directed to the corresponding author.

## Abbreviations

ML, machine learning; AAs, amino acids; AA, amino acid; NASH, non-alcoholic steatohepatitis; IS, internal standard; QC, control samples; UPLC–MS/MS, ultra-high performance liquid chromatography–tandem mass spectrometry; OPLS-DA, orthogonal partial least squares discriminant analysis; VIP, variables of importance in the project; PCA, principal component analysis; LASSO, least absolute shrinkage and selection operator; RF, random forest; KNN, K-nearest neighbor; SVR, support vector regression; XGB, extreme gradient boosting; SHAP, SHapley Additive exPlanations algorithm; MAE, mean absolute error; RMSE, root mean square error; *R* ^2^, coefficient of determination; PH, partial hepatectomy; MS/MS, tandem mass spectrometry; NO, nitric oxide; GSH, glutathione; HE; hematoxylin-eosin; R, Pearson’s correlation coefficient.

## References

[B1] Abu Rmilah A. Zhou W. Nelson E. Lin L. Amiot B. Nyberg S. L. (2019). Understanding the marvels behind liver regeneration. Wiley Interdiscip. Rev. Dev. Biol. 8 (3), e340. 10.1002/wdev.340 30924280 PMC6457252

[B2] Ahmed E. A. Montalti R. Nicolini D. Vincenzi P. Coletta M. Vecchi A. (2016). Fast track program in liver resection: a PRISMA-compliant systematic review and meta-analysis. Med. Baltim. 95 (28), e4154. 10.1097/md.0000000000004154 PMC4956800 27428206

[B3] Ascha M. S. Hanouneh I. A. Lopez R. Tamimi T. A. Feldstein A. F. Zein N. N. (2010). The incidence and risk factors of hepatocellular carcinoma in patients with non-alcoholic steatohepatitis. Hepatology 51 (6), 1972–1978. 10.1002/hep.23527 20209604

[B4] Bifarin O. O. Gaul D. A. Sah S. Arnold R. S. Ogan K. Master V. A. (2021). Urine-based metabolomics and machine learning reveals metabolites associated with renal cell carcinoma stage. Cancers (Basel) 13 (24), 6253. 10.3390/cancers13246253 34944874 PMC8699523

[B5] Boland M. L. Oró D. Tølbøl K. S. Thrane S. T. Nielsen J. C. Cohen T. S. (2019). Towards a standard diet-induced and biopsy-confirmed mouse model of non-alcoholic steatohepatitis: impact of dietary fat source. World J. Gastroenterol. 25 (33), 4904–4920. 10.3748/wjg.v25.i33.4904 31543682 PMC6737317

[B6] Bottiglieri T. Wang X. Arning E. Fernandez H. Wall A. McKenna G. (2022). Longitudinal profiling of plasma and urine metabolites during liver regeneration in living liver donors. Clin. Transpl. 36 (1), e14490. 10.1111/ctr.14490 34545967

[B7] Bratulic S. Limeta A. Dabestani S. Birgisson H. Enblad G. Stålberg K. (2022). Noninvasive detection of any-stage cancer using free glycosaminoglycans. Proc. Natl. Acad. Sci. U. S. A. 119 (50), e2115328119. 10.1073/pnas.2115328119 36469776 PMC9897435

[B8] Canellakis E. S. Kyriakidis D. A. Rinehart C. A. Jr. Huang S. C. Panagiotidis C. Fong W. F. (1985). Regulation of polyamine biosynthesis by antizyme and some recent developments relating the induction of polyamine biosynthesis to cell growth. Review. Biosci. Rep. 5 (3), 189–204. 10.1007/bf01119588 3893559

[B9] Carril E. Valdecantos M. P. Lanzón B. Angulo S. Valverde Á M. Godzien J. (2020). Metabolic impact of partial hepatectomy in the non-alcoholic steatohepatitis animal model of methionine-choline deficient diet. J. Pharm. Biomed. Anal. 178, 112958. 10.1016/j.jpba.2019.112958 31718984

[B10] Dai X. M. Long Z. T. Zhu F. F. Li H. J. Xiang Z. Q. Wu Y. C. (2023). Expression profiles of lncRNAs, miRNAs, and mRNAs during the proliferative phase of liver regeneration in mice with liver fibrosis. Genomics 115 (5), 110707. 10.1016/j.ygeno.2023.110707 37722434

[B11] de Meijer V. E. Kalish B. T. Puder M. Ijzermans J. N. (2010). Systematic review and meta-analysis of steatosis as a risk factor in major hepatic resection. Br. J. Surg. 97 (9), 1331–1339. 10.1002/bjs.7194 20641066

[B12] Deo R. C. (2015). Machine learning in medicine. Circulation 132 (20), 1920–1930. 10.1161/circulationaha.115.001593 26572668 PMC5831252

[B13] Di Minno A. Gelzo M. Caterino M. Costanzo M. Ruoppolo M. Castaldo G. (2022). Challenges in metabolomics-based tests, biomarkers revealed by metabolomic analysis, and the promise of the application of metabolomics in precision medicine. Int. J. Mol. Sci. 23 (9), 5213. 10.3390/ijms23095213 35563604 PMC9103094

[B14] D'Onofrio M. De Robertis R. Demozzi E. Crosara S. Canestrini S. Pozzi Mucelli R. (2014). Liver volumetry: is imaging reliable? Personal experience and review of the literature. World J. Radiol. 6 (4), 62–71. 10.4329/wjr.v6.i4.62 24778768 PMC4000610

[B15] Fujisawa K. Takami T. Okubo S. Nishimura Y. Yamada Y. Kondo K. (2021). Establishment of an adult medaka fatty liver model by administration of a gubra-amylin-non-alcoholic steatohepatitis diet containing high levels of palmitic acid and fructose. Int. J. Mol. Sci. 22 (18), 9931. 10.3390/ijms22189931 34576091 PMC8467182

[B16] Galal A. Talal M. Moustafa A. (2022). Applications of machine learning in metabolomics: disease modeling and classification. Front. Genet. 13, 1017340. 10.3389/fgene.2022.1017340 36506316 PMC9730048

[B17] Gasman S. Chasserot-Golaz S. Popoff M. R. Aunis D. Bader M. F. (1997). Trimeric G proteins control exocytosis in chromaffin cells. Go regulates the peripheral actin network and catecholamine secretion by a mechanism involving the small GTP-binding protein Rho. J. Biol. Chem. 272 (33), 20564–20571. 10.1074/jbc.272.33.20564 9252370

[B18] Geh D. Manas D. M. Reeves H. L. (2021). Hepatocellular carcinoma in non-alcoholic fatty liver disease-a review of an emerging challenge facing clinicians. Hepatobiliary Surg. Nutr. 10 (1), 59–75. 10.21037/hbsn.2019.08.08 33575290 PMC7867726

[B19] Guglielmi A. Ruzzenente A. Conci S. Valdegamberi A. Iacono C. (2012). How much remnant is enough in liver resection? Dig. Surg. 29 (1), 6–17. 10.1159/000335713 22441614

[B20] Handelman G. S. Kok H. K. Chandra R. V. Razavi A. H. Lee M. J. Asadi H. (2018). eDoctor: machine learning and the future of medicine. J. Intern Med. 284 (6), 603–619. 10.1111/joim.12822 30102808

[B21] Huang Z. Z. Li H. Cai J. Kuhlenkamp J. Kaplowitz N. Lu S. C. (1998). Changes in glutathione homeostasis during liver regeneration in the rat. Hepatology 27 (1), 147–153. 10.1002/hep.510270123 9425930

[B22] Irino Y. Toh R. Nagao M. Mori T. Honjo T. Shinohara M. (2016). 2-Aminobutyric acid modulates glutathione homeostasis in the myocardium. Sci. Rep. 6, 36749. 10.1038/srep36749 27827456 PMC5101505

[B23] Ito K. Ozasa H. Noda Y. Koike Y. Arii S. Horikawa S. (2008). Effect of non-essential amino acid glycine administration on the liver regeneration of partially hepatectomized rats with hepatic ischemia/reperfusion injury. Clin. Nutr. 27 (5), 773–780. 10.1016/j.clnu.2008.06.012 18692283

[B24] Ji M. Jo Y. Choi S. J. Kim S. M. Kim K. K. Oh B. C. (2022). Plasma metabolomics and machine learning-driven novel diagnostic signature for non-alcoholic steatohepatitis. Biomedicines 10 (7), 1669. 10.3390/biomedicines10071669 35884973 PMC9312563

[B25] Jung S. Ahn E. Koh S. B. Lee S. H. Hwang G. S. (2021). Purine metabolite-based machine learning models for risk prediction, prognosis, and diagnosis of coronary artery disease. Biomed. Pharmacother. 139, 111621. 10.1016/j.biopha.2021.111621 34243599

[B26] Jung Y. S. (2015). Metabolism of sulfur-containing amino acids in the liver: a link between hepatic injury and recovery. Biol. Pharm. Bull. 38 (7), 971–974. 10.1248/bpb.b15-00244 26133705

[B27] Jung Y. S. Kim S. J. Kwon D. Y. Jun D. S. Kim Y. C. (2013). Significance of alterations in the metabolomics of sulfur-containing amino acids during liver regeneration. Biochimie 95 (8), 1605–1610. 10.1016/j.biochi.2013.04.015 23669448

[B28] Jung Y. S. Kim S. J. Kwon D. Y. Kim Y. C. (2012). Metabolomic analysis of sulfur-containing substances and polyamines in regenerating rat liver. Amino Acids 42 (6), 2095–2102. 10.1007/s00726-011-0946-7 21626405

[B29] Kajiura D. Yamanaka-Okumura H. Hirayama A. Tatano H. Endo K. Honma M. (2019). Perioperative serum and urine metabolome analyses in patients with hepatocellular carcinoma undergoing partial hepatectomy. Nutrition 58, 110–119. 10.1016/j.nut.2018.06.002 30391689

[B30] Konerman M. A. Jones J. C. Harrison S. A. (2018). Pharmacotherapy for NASH: current and emerging. J. Hepatol. 68 (2), 362–375. 10.1016/j.jhep.2017.10.015 29122694

[B31] Lei X. Dai X. Wang Q. Long R. Xiang Z. Li H. (2022). RNA-seq transcriptome profiling of liver regeneration in mice identifies the miR-34b-5p/phosphoinositide-dependent protein kinase 1 axis as a potential target for hepatocyte proliferation. Biochem. Biophys. Res. Commun. 627, 111–121. 10.1016/j.bbrc.2022.08.049 36030652

[B32] Liu W. Baker R. D. Bhatia T. Zhu L. Baker S. S. (2016). Pathogenesis of non-alcoholic steatohepatitis. Cell Mol. Life Sci. 73 (10), 1969–1987. 10.1007/s00018-016-2161-x 26894897 PMC11108381

[B33] Lorente S. Hautefeuille M. Sanchez-Cedillo A. (2020). The liver, a functionalized vascular structure. Sci. Rep. 10 (1), 16194. 10.1038/s41598-020-73208-8 33004881 PMC7531010

[B34] Luciani A. Rusko L. Baranes L. Pichon E. Loze B. Deux J. F. (2012). Automated liver volumetry in orthotopic liver transplantation using multiphase acquisitions on MDCT. AJR Am. J. Roentgenol. 198 (6), W568–W574. 10.2214/ajr.11.7468 22623572

[B35] Lundberg S. Lee S. I. (2017) A unified approach to interpreting model predictions. Nips.

[B36] Mei Y. Thevananther S. (2011). Endothelial nitric oxide synthase is a key mediator of hepatocyte proliferation in response to partial hepatectomy in mice. Hepatology 54 (5), 1777–1789. 10.1002/hep.24560 21748771 PMC3579770

[B37] Michalopoulos G. K. (2017). Hepatostat: liver regeneration and normal liver tissue maintenance. Hepatology 65 (4), 1384–1392. 10.1002/hep.28988 27997988

[B38] Minin E. A. Buchwalow I. B. Wellner M. Palmes D. Spiegel H. U. Neumann J. (2005). L-Arginine-NO-cGMP signaling following acute liver injury in the rat. Exp. Toxicol. Pathol. 57 (2), 161–171. 10.1016/j.etp.2005.08.003 16325526

[B39] Pahlavan P. S. Feldmann R. E. Jr. Zavos C. Kountouras J. (2006). Prometheus' challenge: molecular, cellular and systemic aspects of liver regeneration. J. Surg. Res. 134 (2), 238–251. 10.1016/j.jss.2005.12.011 16458925

[B40] Reddy S. K. Marsh J. W. Varley P. R. Mock B. K. Chopra K. B. Geller D. A. (2012). Underlying steatohepatitis, but not simple hepatic steatosis, increases morbidity after liver resection: a case-control study. Hepatology 56 (6), 2221–2230. 10.1002/hep.25935 22767263

[B41] Sergeeva O. Sviridov E. Zatsepin T. (2020). Noncoding RNA in liver regeneration-from molecular mechanisms to clinical implications. Semin. Liver Dis. 40 (1), 70–83. 10.1055/s-0039-1693513 31323689

[B42] Soyer P. Sirol M. Dohan A. Gayat E. Placé V. Hristova L. (2012). Hepatic height on coronal computed tomography images predicts total liver volume in European adults without liver disease. Dig. Dis. Sci. 57 (6), 1692–1697. 10.1007/s10620-012-2077-8 22314346

[B43] Sun R. Zhao H. Huang S. Zhang R. Lu Z. Li S. (2021). Prediction of liver weight recovery by an integrated metabolomics and machine learning approach after 2/3 partial hepatectomy. Front. Pharmacol. 12, 760474. 10.3389/fphar.2021.760474 34916939 PMC8669962

[B44] Troyanskaya O. Cantor M. Sherlock G. Brown P. Hastie T. Tibshirani R. (2001). Missing value estimation methods for DNA microarrays. Bioinformatics 17 (6), 520–525. 10.1093/bioinformatics/17.6.520 11395428

[B45] Wang Q. Long Z. Zhu F. Li H. Xiang Z. Liang H. (2023). Integrated analysis of lncRNA/circRNA-miRNA-mRNA in the proliferative phase of liver regeneration in mice with liver fibrosis. BMC Genomics 24 (1), 417. 10.1186/s12864-023-09478-z 37488484 PMC10364436

[B46] Wu G. (2009). Amino acids: metabolism, functions, and nutrition. Amino Acids 37 (1), 1–17. 10.1007/s00726-009-0269-0 19301095

[B47] Xu C. S. Chang C. F. (2008). Expression profiles of the genes associated with metabolism and transport of amino acids and their derivatives in rat liver regeneration. Amino Acids 34 (1), 91–102. 10.1007/s00726-007-0576-2 17713745

[B48] Yang H. Guo J. Jin W. Chang C. Guo X. Xu C. (2020). A combined proteomic and metabolomic analyses of the priming phase during rat liver regeneration. Arch. Biochem. Biophys. 693, 108567. 10.1016/j.abb.2020.108567 32898568

[B49] Zamboni G. A. Pedrosa I. Kruskal J. B. Raptopoulos V. (2008). Multimodality postoperative imaging of liver transplantation. Eur. Radiol. 18 (5), 882–891. 10.1007/s00330-007-0840-6 18175119

[B50] Zhao Y. Chen E. Huang K. Xie Z. Zhang S. Wu D. (2020). Dynamic alterations of plasma metabolites in the progression of liver regeneration after partial hepatectomy. J. Proteome Res. 19 (1), 174–185. 10.1021/acs.jproteome.9b00493 31802674

