# Supplementary material for: Amino acid metabolomics and machine learning for assessment of post-hepatectomy liver regeneration
Source: Front Pharmacol. 2024 May 24;15:1345099. doi: 10.3389/fphar.2024.1345099 (PMC11157015; doi:10.3389/fphar.2024.1345099)
Supplement: Supplementary file 1 [file DataSheet2.docx]

***Supplementary Material***

**Supplementary Data Sheet 1** Original data and the importance of amino acids in each model.

**Supplementary Table S1** Multiple reaction monitoring parameter.

**Supplementary Table S2** Differences in liver index between NASH and healthy groups at different time points.

**Supplementary Table S3** Variables of importance in the project analysis and statistical testing.

**Supplementary Table S4** Analysis of amino acid metabolic pathways.

**Supplementary Table S5** The hyperparameters of the optimization models.

**Supplementary Table S6** Models performance.

**Supplementary Table S7** Comparison of five machine learning model’s coefficient of determination, root mean square error, mean absolute error.

**Supplementary Table S8** Correlation between amino acid concentration and liver index.

**Supplementary Figure S1** Changes in the content of the same amino acids in the healthy and NASH groups.

**Supplementary Figure S2** The heatmap shows the Pearson’s correlation coefficients between the liver index and individual amino acids.

**Supplementary Table S1** Multiple reaction monitoring parameter.

| Compound Name | Parent (m/z) | Daughter (m/z) | Cone (V) | Collision (V) | Molecular Weight |
| --- | --- | --- | --- | --- | --- |
| Histidine (His) | 163.76 | 110.05 | 24 | 12 | 155.15 |
| 1-Methyl-L-histidine (1MHis) | 170.62 | 124.02 | 23 | 13 | 169.18 |
| 3-Methyl-L-histidine (3MHis) | 170.87 | 95.88 | 28 | 17 | 169.18 |
| Hydroxyproline (Hyp) | 302.1596 | 171.0844 | 40 | 18 | 131.13 |
| Asparagine (Asn) | 303.0958 | 171.0591 | 38 | 16 | 132.12 |
| Phosphorylethanolamine (PEtN) | 312.0958 | 171.0774 | 25 | 15 | 141.06 |
| Arginine (Arg) | 173.19 | 171.02 | 26 | 12 | 174.2 |
| Carnosine (Car) | 199.21 | 171.06 | 26 | 17 | 226.23 |
| Anserine (Ans) | 206.26 | 171.06 | 29 | 13 | 240.26 |
| Argininosuccinic acid (Asa) | 231.07 | 171.04 | 24 | 14 | 290.27 |
| Serine (Ser) | 276.0958 | 171.0868 | 34 | 16 | 105.09 |
| Taurine (Tau) | 296.0958 | 171.0742 | 26 | 14 | 125.15 |
| Glutamine (Gln) | 317.1503 | 171.0942 | 38 | 20 | 146.14 |
| Ethanolamine (EtN) | 232.0957 | 171.0983 | 38 | 22 | 61.08 |
| Glycine (Gly) | 246.0319 | 171.0716 | 36 | 20 | 75.07 |
| Sarcosine (Sar) | 260.0958 | 171.0838 | 34 | 18 | 89.09 |
| beta-Alanine (bAla) | 260.0958 | 171.0838 | 34 | 18 | 89.09 |
| Threonine (Thr) | 290.1596 | 171.0985 | 36 | 18 | 119.12 |
| Aspartic acid (Asp) | 304.2234 | 171.0747 | 20 | 20 | 133.1 |
| Glutamic acid (Glu) | 318.1596 | 171.1504 | 45 | 24 | 147.13 |
| Citrulline (Cit) | 346.2234 | 171.1387 | 33 | 20 | 175.19 |
| Alanine (Ala) | 260.0958 | 171.0838 | 34 | 18 | 89.09 |

| Compound Name | Parent  (m/z) | Daughter (m/z) | Cone  (V) | Collision (V) | Molecular Weight |
| --- | --- | --- | --- | --- | --- |
| gamma-Aminobutyric acid (GABA) | 274.0958 | 171.0952 | 36 | 20 | 103.12 |
| Aminoisobutyric acid (bAib) | 274.0958 | 171.0952 | 36 | 20 | 103.12 |
| Proline (Pro) | 286.1596 | 171.0797 | 32 | 18 | 115.13 |
| Aminoadipic acid (aAd) | 332.1596 | 171.0664 | 35 | 20 | 161.16 |
| 5-Hydroxylysine (Hyl) | 252.1 | 171.1 | 36 | 15 | 162.19 |
| Homocitrulline (Hcit) | 360.2234 | 171.086 | 37 | 24 | 189.21 |
| 2-Aminobutyric acid (Abu) | 274.0958 | 171.0952 | 36 | 20 | 103.12 |
| Valine (Val) | 288.2873 | 171.0706 | 42 | 20 | 117.15 |
| Methionine (Met) | 320.1596 | 171.0783 | 42 | 22 | 149.21 |
| Tyrosine (Tyr) | 352.2234 | 171.0848 | 36 | 24 | 181.19 |
| Cystathionine (Cth) | 282.1 | 171.1 | 30 | 15 | 222.26 |
| Cystine (Cys) | 291.1 | 171.1 | 30 | 15 | 240.3 |
| Leucine (Leu) | 302.1596 | 171.0844 | 40 | 18 | 131.17 |
| Isoleucine (Ile) | 302.1596 | 171.0844 | 40 | 18 | 131.17 |
| Phenylalanine (Phe) | 336.1596 | 171.0811 | 44 | 22 | 165.19 |
| Tryptophan (Trp) | 375.2234 | 171.077 | 42 | 28 | 204.22 |
| Lysine (Lys) | 244.1 | 171.1 | 20 | 15 | 146.19 |

**Supplementary Table S2** Differences in liver index between NASH and healthy groups at different time points.

| Time points | Statistical test | *p-value* |
| --- | --- | --- |
| 6 h | Independent Samples t-test | 0.235 |
| 24 h | Independent Samples t-test | 0.937 |
| 48 h | Independent Samples t-test | 0.296 |
| 72 h | Independent Samples t-test | 0.211 |
| 168 h | Independent Samples t-test | ＜0.000 |

**Supplementary Table S3A** Variables of importance in the project analysis and statistical testing of the healthy group.

| Amino acids | VIP | Statistical test | Significance |  |
| --- | --- | --- | --- | --- |
| Met | 1.21202 | One-way ANOVA | * |  |
| Asn | 1.19527 | One-way ANOVA | * |  |
| PEtN | 1.18875 | One-way ANOVA | / |  |
| Ans | 1.17363 | One-way ANOVA | / |  |
| Hyl | 1.13863 | Kruskal-Wallis H | * |  |
| Car | 1.13172 | One-way ANOVA | / |  |
| Hcit | 1.11780 | One-way ANOVA | * |  |
| Tyr | 1.09077 | One-way ANOVA | * |  |
| Pro | 1.08893 | Kruskal-Wallis H | * |  |
| 3MHis | 1.07998 | Kruskal-Wallis H | * |  |
| Ala | 1.06531 | One-way ANOVA | * |  |
| Cit | 1.04395 | Kruskal-Wallis H | / |  |
| Hyp | 1.02111 | Kruskal-Wallis H | * |  |
| Ser | 1.01333 | One-way ANOVA | * |  |
| Val | 1.00224 | One-way ANOVA | * |  |
| * indicates significant difference (*p* < 0.05), / indicates no difference. | | | | |

**Supplementary Table S3B** Variables of importance in the project analysis and statistical testing of the NASH group.

| Amino acids | VIP | Statistical test | Significance |
| --- | --- | --- | --- |
| Asa | 1.26755 | One-way ANOVA | * |
| Sar | 1.25507 | Kruskal-Wallis H | * |
| Hyl | 1.15647 | One-way ANOVA | * |
| bAla | 1.14060 | One-way ANOVA | * |
| Arg | 1.13254 | Kruskal-Wallis H | * |
| Hyp | 1.09406 | One-way ANOVA | * |
| Val | 1.08398 | One-way ANOVA | * |
| His | 1.07959 | Kruskal-Wallis H | * |
| Abu | 1.07146 | One-way ANOVA | * |
| Leu | 1.06017 | One-way ANOVA | * |
| 1MHis | 1.05945 | One-way ANOVA | * |
| Tyr | 1.05585 | One-way ANOVA | * |
| Ile | 1.04663 | One-way ANOVA | * |
| Asn | 1.04634 | One-way ANOVA | * |
| GABA | 1.04084 | One-way ANOVA | * |
| Asp | 1.03492 | One-way ANOVA | * |
| Cys | 1.02024 | Kruskal-Wallis H | * |
| Tau | 1.01944 | One-way ANOVA | * |
| Gly | 1.01842 | One-way ANOVA | * |
| Gln | 1.01541 | One-way ANOVA | * |
| EtN | 1.00831 | Kruskal-Wallis H | * |
| Thr | 1.00132 | One-way ANOVA | * |
| *indicates significant difference (*p* < 0.05). | | | |

**Supplementary Table S4A** Pathway results of the healthy group.

| Pathway name | Match  status | Raw p | -log(p) | Impact |
| --- | --- | --- | --- | --- |
| Arg and Pro metabolism | 2/38 | 0.019964 | 1.6997 | 0.13879 |
| Phe, Tyr and Trp biosynthesis | 1/4 | 0.023714 | 1.625 | 0.5 |

**Supplementary Table S4B** Pathway results of the NASH group.

| Pathway name | Match  status | Raw p | -log(p) | Impact |
| --- | --- | --- | --- | --- |
| Arg biosynthesis | 4/14 | 0.000031148 | 4.5066 | 0.19289 |
| Ala, Asp and glutamate metabolism | 5/28 | 0.000032426 | 4.4891 | 0.44551 |
| Gly, Ser and Thr metabolism | 4/34 | 0.0011896 | 2.9246 | 0.38193 |
| His metabolism | 3/16 | 0.0013412 | 2.8725 | 0.22131 |
| bAla metabolism | 3/21 | 0.0030375 | 2.5175 | 0.39925 |
| Tau and hypotaurine metabolism | 2/8 | 0.0054109 | 2.2667 | 0.42857 |
| Arg and Pro metabolism | 3/38 | 0.016396 | 1.7853 | 0.1427 |

**Supplementary Table S5A.** The best-tuned hyperparameters for each model in the healthy group.

| Model | Hyperparameter | Optimal value |
| --- | --- | --- |
| LASSO | Lambda | 1.1895e-04 |
| RF | N estimators | 34 |
|  | Max depth | 8 |
|  | Max features | 5 |
|  | Min samples leaf | 1 |
|  | Min samples split | 5 |
| KNN | N neighbors | 6 |
|  | Weights | distance |
|  | P | 1 |
| XGB | N estimators | 58 |
|  | Max depth | 3 |
|  | Max leaves | 1 |
|  | Subsample | 0.9 |
|  | Booster | dart |
| SVR | Kernel | rbf |
|  | Epsilon | 8e-05 |
|  | Gamma | 1.8 |
|  | C | 0.02 |

LASSO, least absolute shrinkage and selection operator; RF, random forest; KNN, K-nearest neighbor; XGB, extreme gradient boosting; SVR, support vector regression.

**Supplementary Table S5B.** The best-tuned hyperparameters for each model in the NASH group.

| Model | Hyperparameter | Optimal value |
| --- | --- | --- |
| LASSO | Lambda | 6.4424e-06 |
| RF | N estimators | 109 |
|  | Max depth | 7 |
|  | Max features | 4 |
|  | Min samples leaf | 1 |
|  | Min samples split | 2 |
| KNN | N neighbors | 6 |
|  | Weights | uniform |
|  | P | 1 |
| XGB | N estimators | 120 |
|  | Max depth | 3 |
|  | Max leaves | 1 |
|  | Subsample | 0.85 |
|  | Booster | dart |
| SVR | Kernel | rbf |
|  | Epsilon | 0.0011 |
|  | Gamma | 0.77 |
|  | C | 0.009 |

LASSO, least absolute shrinkage and selection operator; RF, random forest; KNN, K-nearest neighbor; XGB, extreme gradient boosting; SVR, support vector regression.

**Supplementary Table S6A** Five models performance of the healthy group.

| Model | R^2^ | RMSE | MAE |
| --- | --- | --- | --- |
| LASSO | 0.66 ± 0.23 | 0.0061 ± 0.0014 | 0.0048 ± 0.0011 |
| RF | 0.78 ± 0.19 | 0.0047 ± 0.0015 | 0.0038 ± 0.0011 |
| KNN | 0.79 ± 0.17 | 0.0047 ± 0.0013 | 0.0037 ± 0.0010 |
| XGB | 0.78 ± 0.14 | 0.0049 ± 0.0015 | 0.0039 ± 0.0012 |
| SVR | 0.78 ± 0.14 | 0.0049 ± 0.0012 | 0.0036 ± 0.0009 |

Values are presented as mean ± SD. R^2^, coefficient of determination; RMSE, root mean square error; MAE, mean absolute error; LASSO, least absolute shrinkage and selection operator; RF, random forest; KNN, K-nearest neighbor; XGB, extreme gradient boosting; SVR, support vector regression.

**Supplementary Table S6B** Five models performance of the NASH group.

| Model | R^2^ | RMSE | MAE |
| --- | --- | --- | --- |
| LASSO | 0.74 ± 0.19 | 0.0031 ± 0.0007 | 0.0026 ± 0.0007 |
| RF | 0.68 ± 0.17 | 0.0036 ± 0.0008 | 0.0029 ± 0.0007 |
| KNN | 0.71 ± 0.18 | 0.0034 ± 0.0009 | 0.0028 ± 0.0006 |
| XGB | 0.69 ± 0.19 | 0.0034 ± 0.0009 | 0.0028 ± 0.0007 |
| SVR | 0.69 ± 0.21 | 0.0034 ± 0.0007 | 0.0029 ± 0.0006 |

Values are presented as mean ± SD. R^2^, coefficient of determination; RMSE, root mean square error; MAE, mean absolute error; LASSO, least absolute shrinkage and selection operator; RF, random forest; KNN, K-nearest neighbor; XGB, extreme gradient boosting; SVR, support vector regression.

**Supplementary Table S7A.** Comparison of five models coefficient of determination in the healthy group.

|  | LASSO | RF | KNN | XGB | SVR |
| --- | --- | --- | --- | --- | --- |
| LASSO | \ | 0.000021 | 0.000029 | 0.000390 | 0.000319 |
| RF | 0.000021 | \ | 0.945976 | 0.482366 | 0.515723 |
| KNN | 0.000029 | 0.945976 | \ | 0.525597 | 0.560438 |
| XGB | 0.000390 | 0.482366 | 0.525597 | \ | 0.958091 |
| SVR | 0.000319 | 0.515723 | 0.560438 | 0.958091 | \ |

LASSO, least absolute shrinkage and selection operator; RF, random forest; KNN, K-nearest neighbor; XGB, extreme gradient boosting; SVR, support vector regression.

**Supplementary Table S7B.** Comparison of five models root mean square error in the healthy group.

|  | LASSO | RF | KNN | XGB | SVR |
| --- | --- | --- | --- | --- | --- |
| LASSO | \ | 0.000003 | 0.000011 | 0.000057 | 0.000070 |
| RF | 0.000003 | \ | 0.806635 | 0.536479 | 0.504178 |
| KNN | 0.000011 | 0.806635 | \ | 0.708868 | 0.672178 |
| XGB | 0.000057 | 0.536479 | 0.708868 | \ | 0.960295 |
| SVR | 0.000070 | 0.504178 | 0.672178 | 0.960295 | \ |

LASSO, least absolute shrinkage and selection operator; RF, random forest; KNN, K-nearest neighbor; XGB, extreme gradient boosting; SVR, support vector regression.**Supplementary Table S7C.** Comparison of five models mean absolute error in the healthy group.

|  | LASSO | RF | KNN | XGB | SVR |
| --- | --- | --- | --- | --- | --- |
| LASSO | \ | 0.000017 | 0.000007 | 0.000505 | 0.000002 |
| RF | 0.000017 | \ | 0.839995 | 0.409829 | 0.662119 |
| KNN | 0.000007 | 0.839995 | \ | 0.304847 | 0.814140 |
| XGB | 0.000505 | 0.409829 | 0.304847 | \ | 0.207242 |
| SVR | 0.000002 | 0.662119 | 0.814140 | 0.207242 | \ |

LASSO, least absolute shrinkage and selection operator; RF, random forest; KNN, K-nearest neighbor; XGB, extreme gradient boosting; SVR, support vector regression.

**Supplementary Table S7D.** Comparison of five model’s coefficient of determination, root mean square error, mean absolute error in the NASH group.

|  | R^2^ | RMSE | MAE |
| --- | --- | --- | --- |
| Kruskal–Wallis Test | 5.936 | 8.560 | 7.707 |
| *p* | 0.204 | 0.073 | 0.103 |

R^2^, coefficient of determination; RMSE, root mean square error; MAE, mean absolute error.

**Supplementary Table S8A** Correlation between amino acid concentration and liver index in the healthy group.

| Amino acids | R | *p-value* | |
| --- | --- | --- | --- |
| Ser | -0.73348 | | 3.60E-07 |
| Tyr | -0.66023 | | 0.000012 |
| 3MHis | -0.59086 | | 0.000148 |
| Val | -0.52784 | | 0.000938 |
| Hyl | 0.45893 | | 0.004873 |
| Pro | -0.32105 | | 0.056233 |
| Cit | -0.26063 | | 0.124718 |
| Ala | -0.24994 | | 0.141513 |
| Asn | -0.22894 | | 0.179254 |
| Hcit | 0.21504 | | 0.207839 |
| PEtN | -0.21092 | | 0.216895 |
| Hyp | -0.20921 | | 0.220737 |
| Met | -0.19643 | | 0.250888 |
| Ans | -0.11731 | | 0.495635 |
| Car | -0.11235 | | 0.514159 |

R, Pearson's correlation coefficient.

**Supplementary Table S8B** Correlation between amino acid concentration and liver index in the NASH group.

| Amino acids | R | *p-value* |
| --- | --- | --- |
| Arg | 0.81571 | 1.36E-09 |
| EtN | -0.71469 | 9.76E-07 |
| Sar | -0.63534 | 0.000031 |
| Cys | -0.61591 | 0.000064 |
| His | -0.59903 | 0.000113 |
| Gly | -0.59102 | 0.000147 |
| bAla | -0.57157 | 0.000271 |
| Tau | -0.55652 | 0.000424 |
| Asp | -0.4973 | 0.002031 |
| Thr | -0.49348 | 0.002225 |
| Gln | -0.47987 | 0.00306 |
| GABA | -0.41777 | 0.011236 |
| Asa | 0.40492 | 0.014295 |
| Abu | -0.38731 | 0.019607 |
| Leu | -0.37404 | 0.024618 |
| 1MHis | -0.27942 | 0.098856 |
| Hyp | 0.23488 | 0.167918 |
| Tyr | 0.22357 | 0.189952 |
| Ile | 0.12331 | 0.473685 |
| Val | 0.10282 | 0.550698 |
| Asn | 0.033268 | 0.847258 |
| Hyl | -0.01641 | 0.924342 |

R, Pearson's correlation coefficient.


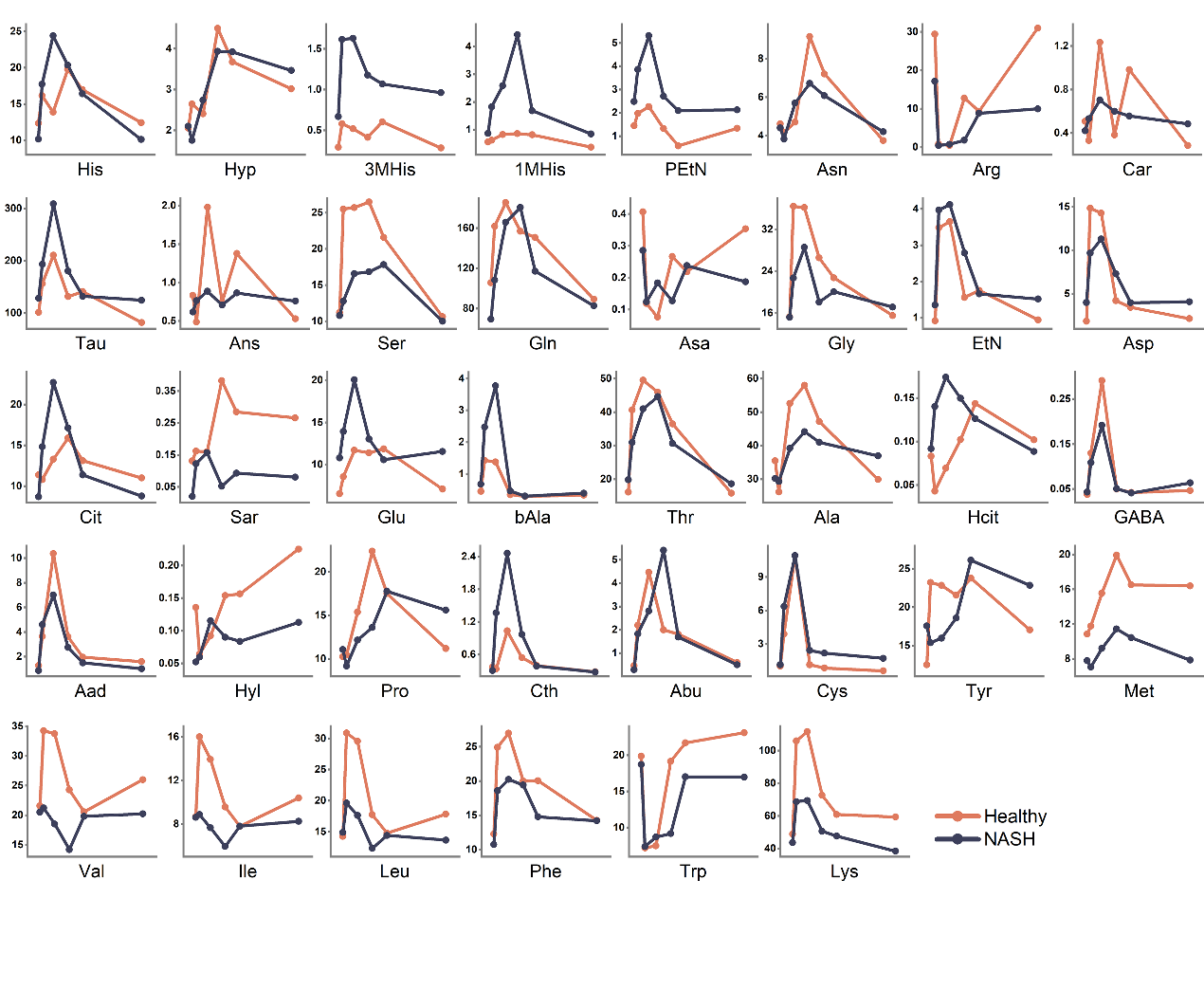


**Supplementary Figure S1** Changes in the content of the same amino acids in the healthy and NASH groups. Vertical axis indicates concentration (μg/ml). The horizontal axis is the time point of serum collection (sham, 6h, 24h, 48h, 72h, 168h).


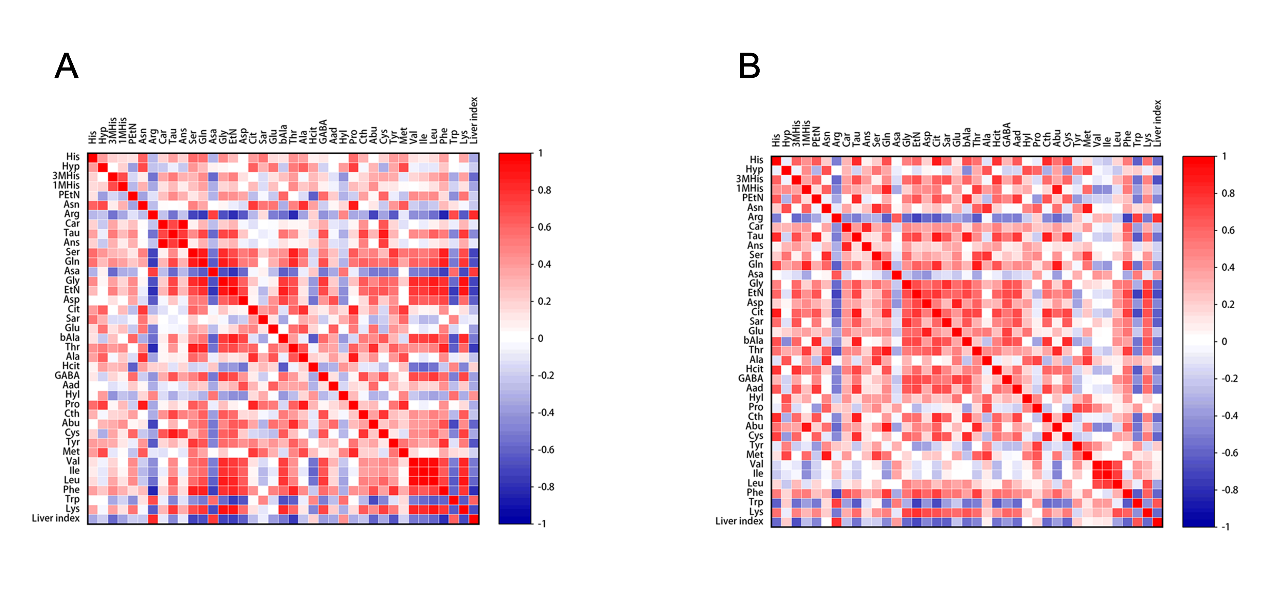
**Supplementary Figure S2** The heatmap shows the Pearson’s correlation coefficients between the liver index and individual amino acids. The magenta cube represents a positive correlation, and the blue cube represents a negative correlation. (A) Healthy group, (B) NASH group.
